# Neurobiomarkers for Traumatic Brain Injury: Comparison of Serum Values Within 24 Hours of Injury With Glasgow Coma Scale (GCS) Scores in a Prospective Cohort Trial

**DOI:** 10.7759/cureus.85152

**Published:** 2025-05-31

**Authors:** Shalini Pasupuleti, Ashima Sharma, Vamsi Krishna Yerramneni, Ramnath Reddy

**Affiliations:** 1 Emergency Medicine, Nizam's Institute of Medical Sciences, Hyderabad, IND; 2 Neurosurgery, Nizam's Institute of Medical Sciences, Hyderabad, IND

**Keywords:** computerized tomography (ct) scan, glasgow coma scale (gcs), neurofilament light (nfl), non-survivors, s100 calcium-binding protein b (s100b), serum biomarkers, traumatic brain injury (tbi)

## Abstract

Objective

Traumatic brain injury (TBI) is a significant cause of morbidity, disability, and mortality across all age groups, presenting both health and socioeconomic challenges globally. Neuroimaging techniques are crucial for assessing TBI, but their availability is often limited. Investing in point-of-care blood biomarkers, such as neurofilament light (NFL) protein and S100 calcium-binding protein B (S100B), may offer more accessible and reliable information on neuronal injury, assisting in clinical evaluation without compromising sensitivity. The objective of this study was to correlate the serum values of NFL and S100B with the severity of TBI as assessed by the Glasgow coma scale and to evaluate the potential of these markers for early prognostication.

Methods

A total of 92 TBI patients, categorized into mild (30), moderate (28), and severe (34) cases, admitted to Nizam’s Institute of Medical Sciences, Hyderabad, India, from 2019 to 2020, were enrolled. Serum levels of NFL and S100B were measured within 24-36 hours of injury for all participants.

Results

NFL concentrations were 51.1±12.13, 99.9±31.55, and 251.68±78.28 pg/mL for mild, moderate, and severe TBI patients, respectively. S100B concentrations were 193.47±76.57, 542.9±158.78, and 1882.6±824.8 pg/mL for mild, moderate, and severe TBI patients, respectively. Significant differences were observed in NFL and S100B levels when compared between the groups (p<0.05). On day 0, the values of NFL (p≤0.001) and S100B (p=0.023) were significantly higher in non-survivors compared to survivors in severe TBI. S100B showed AUCs of 0.98 (mild), 0.93 (moderate), and 0.99 (severe); NFL showed AUCs of 0.21, 0.51, and 0.28, respectively. The odds ratio (OR) for S100B in mild TBI was 1.07 (95% CI: 1.00-1.14); all other ORs were close to 1 with 95% CI including 1.

Conclusion

S100B showed strong diagnostic performance across TBI severities, while NFL demonstrated limited utility based on low AUC values and non-significant ORs. These findings support the use of S100B as a more reliable biomarker for TBI assessment.

## Introduction

Traumatic brain injury (TBI) is a significant cause of morbidity, disability, and mortality across all age groups [1]. The Global Burden of Disease Study indicates that TBI resulting from road traffic accidents (RTA) constitutes 40-60% of cases, while 20-30% of injuries presented in emergency departments (ED) occur due to accidents at or near home, such as falls or domestic violence [2].

Head injuries are commonly classified based on the Glasgow Coma Scale (GCS): mild (GCS 14-15), moderate (GCS 9-13), and severe (GCS 3-8). This scale assesses a patient's level of consciousness by evaluating eye, verbal, and motor responses. Ongoing assessment of the GCS is essential for monitoring the patient's condition and documenting disease progression [3]. Neuroimaging further informs patient status, with computed tomography (CT) scans being the primary tool for evaluating brain parenchymal injuries [4]. However, CT scans demonstrate low sensitivity for detecting lower grades of diffuse axonal injuries [5]. In contrast, magnetic resonance imaging (MRI) offers improved visualization of cortical contusions, diffuse axonal shear injuries, and white matter lesions, although its availability and practicality during the acute phase are often limited [6].

According to the Scandinavian Guidelines, head injuries that are moderate, mild (high risk), and mild (medium risk) are referred for CT. Injuries that are deemed mild (low risk) are triaged by the examination of S100 calcium-binding protein B (S100B) levels (if the injury is within 6 h), and if S100B is elevated, they are sent for head CT [7].

The integration of blood biomarkers may enhance the reliability of assessing neuronal injury, aiding clinical evaluation without compromising sensitivity [8]. These biomarkers could serve as cost-effective tools with good specificity for acute assessments, particularly when there are no significant risk factors for intracranial injuries or cases of minor TBI [9].

Early prognostication in TBI is essential, particularly in low-resource settings and in contexts with prolonged organ transplant waiting times. While a variety of blood-based neurobiomarkers are commercially available, we selected S100B and neurofilament light (NFL) for this study due to their ready availability at our institution. Both biomarkers are widely used to detect and evaluate the severity of nervous system damage, especially in brain injuries and neurological disorders. S100B, released by damaged astrocytes, serves as an indicator of ongoing neural distress and has been associated with poor clinical outcomes, including brain death and increased mortality risk [10,11].

## Materials and methods

Aim and objective of the study

The aim and objectives of the study are to assess the correlation between NFL protein and S100B with the severity of TBI in patients and to compare the initial levels of NFL and S100B with the Glasgow Coma Scale (GCS) scores of TBI patients.

Materials and methods

Our study was a prospective observational cohort study. The study was carried out at the Emergency Medicine Department, Nizam's Institute of Medical Sciences (NIMS), Hyderabad, Telangana from January 2019 to March 2020 after obtaining approval from the Institutional Ethics Committee (EC/NIMS/2306/2019). Written informed consent for participation was obtained from all legal guardians of participants. The subjects of either gender aged 18 to 60 years old who presented to the level 1 trauma center after head trauma were included in the study. The patients were segregated into three groups based on GCS score as mild TBI (GCS score of 14-15), moderate TBI (GCS score of 9-13), and severe TBI (GCS score of 3-8) [12]. The control group was recruited and consisted of age- and sex-matched healthy individuals with no history of neurological disorders, head trauma, or systemic illness. Participants were enrolled from the general population and hospital staff volunteers, following the provision of written informed consent. Pregnant women were excluded due to physiological changes in the body that may alter certain biomarker levels, and polytrauma patients were excluded as they may present with a complex clinical picture, making it difficult to isolate the specific effect of TBI on biomarker levels.

Emergency patient care

Brain trauma foundation guidelines were followed in the early stabilization of patients [13]. Every patient received emergency management based on Advanced Trauma Life Support (ATLS) guidelines for neurotrauma. An Initial Airway, Breathing, Circulation (ABC) approach was done. Baseline GCS was documented. If required, the patient was intubated for airway protection and mechanically ventilated for intracranial pressure (ICP) reduction and support of breathing. A non-contrast CT scan was performed within 30 minutes of arrival at the ED. Based on the initial CT report, a neurosurgical opinion was sought to determine the need for surgical intervention or conservative neurocritical management. Further management of the patient was guided by recommended neurocritical care guidelines [14].

Sample collection and processing

Blood samples were obtained by venipuncture into gel separator tubes for serum collection and were centrifuged within 20 to 60 minutes. The serum was then divided into aliquots and stored at −80°C until biochemical analysis. Samples were collected from all groups of TBI patients within 24-36 hours of injury, as well as from 30 healthy age- and sex-matched controls. All controls were screened to confirm the absence of any history of head injury, neurological or psychiatric conditions, or use of medications that could influence biomarker levels. Serum NFL concentrations were measured using MyBiosource ELISA kits with a sensitivity of 10 pg/mL, while S100B concentrations were measured using Elabscience ELISA kits with a sensitivity of 18.75 pg/mL.

Primary outcome

The primary outcome of the study is to evaluate the effectiveness of blood-based biomarkers, specifically NFL and S100B, in predicting clinical outcomes and facilitating early diagnosis in patients with TBI.

Secondary outcome

The secondary outcome involves assessing the feasibility and clinical practicality of utilizing blood-based biomarkers as alternatives to cerebrospinal fluid (CSF)-based markers. Blood-based biomarkers offer a less invasive and more accessible option for clinical application. The study also explores the potential for integrating these biomarkers into routine clinical assessments for TBI, highlighting the importance of translational research in advancing their use as standard diagnostic and prognostic tools.

Statistical analysis

Statistical analysis was performed using SPSS version 19.0 (IBM Corp, Armonk, NY). Parametric data are presented as mean ± standard deviation (SD), while non-parametric data are expressed as median (range). Repeated measures ANOVA was used to compare GCS scores between groups. One-way ANOVA was applied to compare serum biomarker levels across different TBI severity groups (mild, moderate, and severe). An independent sample t-test was used to compare serum biomarkers between survivors and non-survivors. The results are expressed as mean ± SD. A p-value of less than 0.05 was considered statistically significant.

## Results

A total of 1319 TBI victims presented to our ED during the study period. Of these, only 166 patients met the inclusion criteria, primarily based on age and presentation to the ED within 24-36 hours of injury. Ninety-two patients provided informed consent and were categorized based on their GCS score on arrival into three groups: 30 with mild, 28 with moderate, and 34 with severe TBI. The mean ages of the patients in these groups were 38.96±15.22, 39.53±18.70, and 32.09±10.76 years, respectively. The demographic and clinical characteristics of the TBI subjects and healthy controls are presented in Table 1.

**Table 1:** Demographic characteristics of TBI subjects and healthy controls NIMS, Nizam's Institute of Medical Sciences; TBI, traumatic brain injury

Variables	Mild TBI (GCS=14-15), N=30		Moderate TBI (GCS=9-13), N=28	Severe TBI (GCS= 3-8), N=34	Healthy controls, N=30
Age (in years), mean (SD)	38.96±15.22		39.53±18.70	32.09±10.76	36.7±10.89
Male (%)	25 (83.3%)		26 (92.8%)	33 (97.05%)	11 (36.66%)
Female (%)	5 (16.6%)		2 (7.14%)	1 (2.94%)	19 (63.33%)
Mechanism of trauma					
Motor vehicle accident, n (%)		23 (76)	24 (85.7)	29 (85.2)	NA
Fall, n (%)		5 (16.6)	2 (7.1)	4 (11.7)	
Violence, n (%)		2 (6.6)	2 (7.1)	1 (2.9)	
First hospital of contact					
NIMS, n (%)	5 (16.6)		4 (14.2)	9 (26.4)	NA
Referral cases, n (%)	25 (83.3)		24 (85.7)	25 (73.5)	
Days of hospital stay, mean±SD	4.85±2.1		10.35±3.1	13.91±3.31	
Length of ICU stay, mean ± SD (measured in days)	2.5±1.3		6.6±2.04	8.65±2.4	
Length of stay on ventilator, mean ± SD (measured in days)	0.16±0.64		6.34±0.24	7.14±3.06	
Mortality (n)	0		0	11	

Normal healthy adults will have a serum level of less than 100 pg/mL for S100B [15] and 2.8 to 21.4 pg/mL for NFL [16], respectively. Table 2 clearly demonstrates a 100%, 500%, and 1800% increase in S100B levels in mild, moderate, and severe TBI despite a mean sampling delay of 12.8 hours after injury. As all values are statistically significant, we can infer that S100B is a reliable neurobiomarker to classify the severity of TBI patients. In Table 3, NFL values also show a 900%, 1900%, and 4900% increase, respectively, in the three groups of TBI compared to the normal serum levels. This means that the NFL also has the potential to classify the severity of TBI. The mean values of healthy controls in our study were: NFL - 5.04 ± 2.85 pg/mL and S100B - 0.88 ± 0.21 pg/mL. The mean levels of S100B and NFL in severe TBI cases were compared between survivors and non-survivors, as shown in Tables 2 and Table 3. No patient with mild to moderate TBI died.

**Table 2:** S100B between survivors and non-survivors Since there were no deaths among patients with mild and moderate TBI, the p-value, t-value, and df could not be calculated. Among patients with severe TBI, 23 survived and 11 did not survive. The statistical analysis yielded a p-value of 0.023, a t-value of -2.4, and a df of 28.02. GCS, Glasgow Coma Scale; N, number of patients; df, degrees of freedom; TBI, traumatic brain injury; S100B, S100 calcium-binding protein B

GCS categories	Survivors		Non-survivors		P-value	t-value	df-value
	N	S100B (pg/mL)	N	S100B (pg/mL)			
Mild TBI	30	193.47±76.57	0	-	NA	NA	NA
Moderate TBI	28	542.9±158.78	0	-	NA	NA	NA
severe TBI	23	1756.45±544.8	11	2137.38±367.32	0.023	-2.4	-28.02

**Table 3:** NFL between survivors and non-survivors Since there were no deaths among patients with mild and moderate TBI, the p-value, t-value, and df could not be calculated. Among patients with severe TBI, 23 survived and 11 did not survive. The statistical analysis yielded a p-value of <0.001, a t-value of -4.02, and df of 26.12. GCS, Glasgow Coma Scale; N, number of patients; df, degrees of freedom; TBI, traumatic brain injury; NFL, neurofilament light

GCS categories	Survivors		Non-survivors		P-value	t-value	df-value
	N	NFL (pg/mL)	N	NFL (pg/mL)			
Mild TBI	30	51.1±12.13	0	-	NA	NA	NA
Moderate TBI	28	99.9±31.55	0	-	NA	NA	NA
Severe TBI	23	233.74±56.33	11	302.75±41.56	<0.001	-4.02	26.12

**Figure 1:**
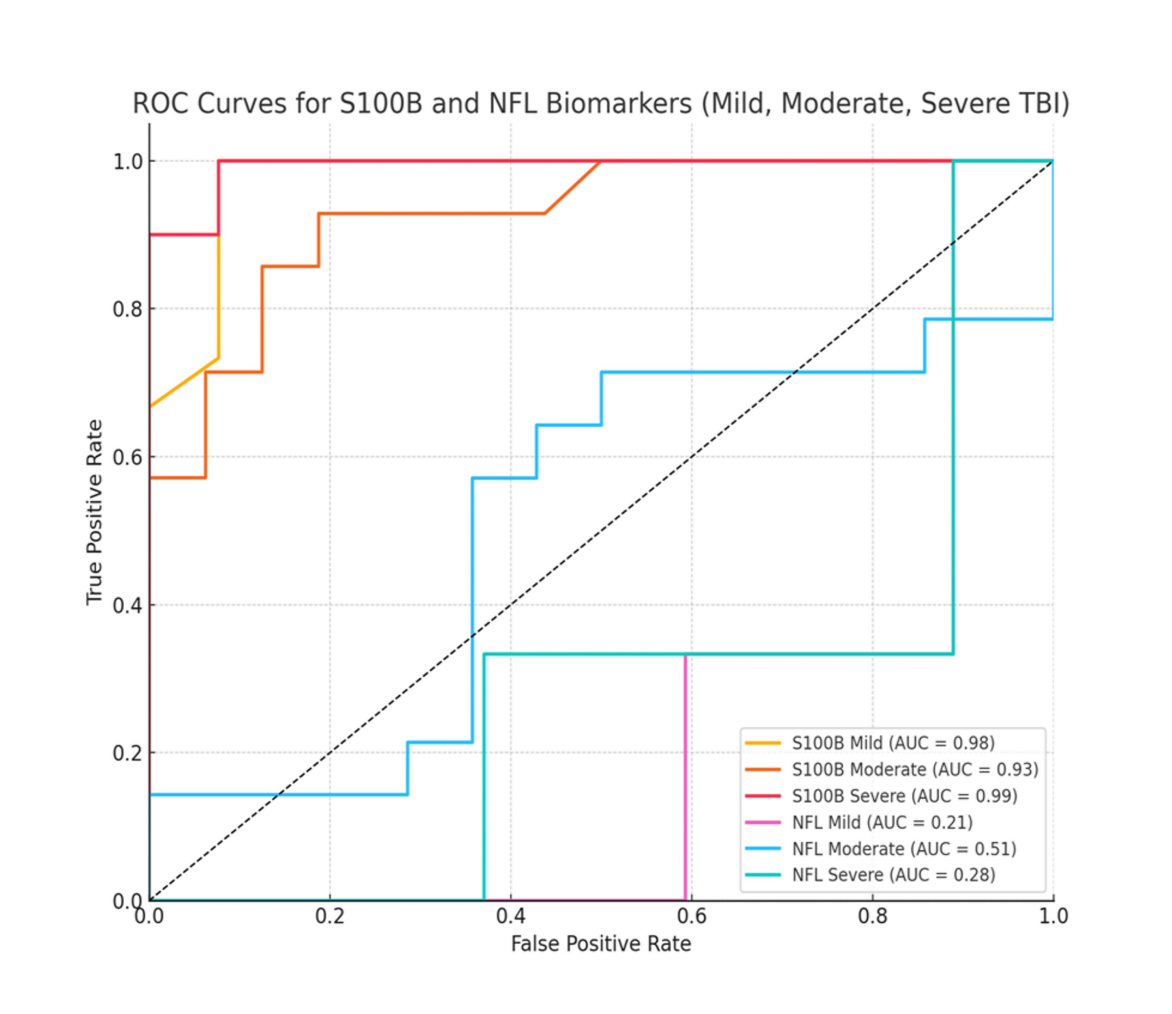
ROC curve showing the AUC for S100B and NFL in assessing diagnostic accuracy ROC curves illustrate the diagnostic performance of S100B and NFL biomarkers across mild, moderate, and severe TBI. S100B demonstrated high diagnostic accuracy (AUC: 0.98-1.00), whereas NFL showed poor discriminative ability (AUC: 0.21-0.51). The dashed line represents the chance level (AUC=0.5). ROC, receiver operating characteristic; NFL, neurofilament light; S100B, S100 calcium-binding protein B; TBI, traumatic brain injury

The ROC plot demonstrates that S100B exhibited excellent diagnostic performance across all TBI severities, with AUC values of 0.98 (mild), 0.93 (moderate), and 1.00 (severe). In contrast, NFL showed poor discriminatory ability, with AUCs of 0.21 (mild), 0.51 (moderate), and 0.28 (severe), indicating limited utility in distinguishing TBI severity (Figure 1).

## Discussion

Over the years, there have been few significant advancements in the clinical assessment of head injuries, with most research focusing on therapeutic interventions during both the early and delayed phases of TBI. Brain-specific blood-based biomarkers present a promising avenue due to their strong translational potential in patient care and management. One of the key challenges remains the development of an efficient medium for early monitoring and outcome prediction. In this context, biomarkers could greatly assist in the diagnosis and prognosis of TBI, highlighting the urgent need for their integration into clinical practice.

While CSF analysis remains the gold standard for evaluating axonal damage, blood-based biomarkers like NFL chains offer a more practical and accessible alternative, especially in cases of mild TBI. Studies have shown a strong correlation between serum and CSF NFL levels, supporting its reliability as an indicator of axonal injury. Compared to other biomarkers such as S100B, which is released from damaged astrocytes and primarily used in emergency settings to help rule out mild TBI, NFL is considered more sensitive and has greater diagnostic and prognostic value [17]. Neurofilaments are intermediate filaments found in the cytoplasm of neurons, and elevated levels of NFL are typically observed within the first seven to 12 days following injury [18]. In mild TBI patients, serum levels of hyperphosphorylated neurofilament H (p-NFH) significantly increase on days one and three [19]. However, a six-hour delay between the onset of injury and the rise in p-NFH blood levels may limit its utility in the acute phase of diagnosis [20]. Elevated NFL levels have been consistently observed in TBI patients when compared to those without TBI, and NFL has shown promise in differentiating between survivors and non-survivors. Initial NFL levels can even predict adverse clinical outcomes up to 12 months following the injury [21]. Few studies have shown that patients with TBI exhibit significantly elevated NFL concentrations compared to healthy controls, persisting for up to five years. Higher NFL levels are associated with moderate to severe TBI cases [22]. Some studies have reported that patients with neurodegenerative diseases such as Alzheimer's disease and Amyotrophic lateral sclerosis (ALS) exhibit elevated serum and CSF NFL levels compared to healthy controls. Additionally, factors such as age, renal insufficiency, diabetes, hypertension, and stroke may also influence NFL levels [23].

S100B is a calcium-binding protein primarily found in astroglial cells in the brain. It has a half-life of 60 to 120 minutes [24]. Increased levels of S100B are associated with brain injury and the increased permeability of the blood-brain barrier. In patients with mild head injuries, S100B levels peak immediately after the trauma but decline sharply, reaching below detection limits (0.0002 μg/L) within six hours due to its short half-life of approximately 30 minutes [25]. In contrast, in severe TBI cases, S100B levels vary with lower levels correlating with with favorable outcomes and higher levels indicating poor prognosis [26]. Elevated serum levels of S100B have been shown to distinguish between survivors and non-survivors of TBI when measured 48 hours after injury [27]. For optimal analysis, S100B should be tested within six hours of injury. However, some studies suggest that sampling within 12 hours post-trauma offers limited prognostic value, with better predictions achieved through serial sampling over 12 to 36 hours. Despite its potential, S100B has limitations due to its presence in extracerebral tissues such as muscle and adipose tissue. IIts sensitivity (61%) and specificity (77%) are relatively low, as they can also be elevated in patients with fractures or other extracranial injuries [28], as well as in conditions such as stroke, neurodegenerative diseases, epilepsy, psychiatric disorders, and melanoma. S100B is also produced in other tissues, including the liver, heart, and kidneys [29]. Interestingly, serum S100B levels have been found to increase in marathon runners without any evidence of brain injury, further complicating its specificity to brain injury in TBI [30]. 

The diagnostic utility of S100B and NFL biomarkers across varying severities of TBI was evaluated through AUC and OR analyses. S100B exhibited excellent discriminatory performance, with AUC values of 0.98, 0.93, and 0.99 for mild, moderate, and severe TBI, respectively, highlighting its strong potential as a diagnostic biomarker. In contrast, NFL demonstrated limited diagnostic value, with notably lower AUCs of 0.21, 0.51, and 0.28 across the same categories. The OR for S100B in mild TBI was 1.07 (95% CI: 1.00-1.14), suggesting a possible association with injury severity. However, for all other biomarkers and TBI severities, the ORs were near unity with CIs encompassing 1, indicating a lack of statistically significant association between biomarker concentrations and clinical outcomes (Table 4).

**Table 4:** ORs with 95% CI for S100B and NFL biomarkers across GCS categories The table presents ORs with 95% CI for the association between biomarker levels and TBI outcomes. An OR greater than 1 indicates a positive association, while an OR less than 1 indicates a negative association. CIs that include 1 suggest that the association is not statistically significant. ORs, odds ratios; NFL, neurofilament light; S100B, S100 calcium-binding protein B; TBI, traumatic brain injury; GCS, Glasgow Coma Scale

Test	OR (95% CI)
S100B mild	1.07 (1.00-1.14)
S100B moderate	1.00 (1.00-1.00)
S100B severe	1.01 (0.99-1.03)
NFL mild	0.93 (0.86-1.02)
NFL moderate	1.00 (0.98-1.03)
NFL severe	0.94 (0.84-1.04)

A key strength of the study lies in its inclusion of TBI cases across all severity levels, providing a comprehensive overview of biomarker behavior throughout the full clinical spectrum. Additionally, biomarker samples were collected within defined time windows, enabling an evaluation of temporal dynamics and the relevance of early versus delayed sampling.

However, a weakness of the study was that only a few patients were directly admitted to NIMS, which restricted control over initial clinical assessments and immediate sample collection protocols.

The limitations of the study include its single-center design, a relatively small sample size, and the absence of long-term follow-up to validate the findings over time.

## Conclusions

Our findings indicate that S100B demonstrates strong diagnostic utility across all severities of TBI, supported by high AUC values (0.98, 0.93, and 0.99 for mild, moderate, and severe TBI, respectively) and a suggestive OR in mild TBI (OR: 1.07; 95% CI: 1.00-1.14). In contrast, NFL showed consistently low AUC values (0.21-0.51) and ORs close to 1 with CIs crossing unity, reflecting limited diagnostic value. These results support the clinical relevance of S100B over NFL in the early assessment and stratification of TBI. Further large-scale, prospective studies are warranted to validate these biomarkers and refine their role in routine TBI evaluation.
